# Hazardous volcanic CO_2_ diffuse degassing areas – A systematic review on environmental impacts, health, and mitigation strategies

**DOI:** 10.1016/j.isci.2024.110990

**Published:** 2024-09-19

**Authors:** Fátima Viveiros, Catarina Silva

**Affiliations:** 1 Instituto de Investigação em Vulcanologia e Avaliação de Riscos (IVAR), Universidade dos Açores, Rua Mãe de Deus, 9500-801 Ponta Delgada, Azores, Portugal; 2 Faculdade de Ciências e Tecnologia (FCT), Universidade dos Açores, Rua Mãe de Deus, 9500-801 Ponta Delgada, Azores, Portugal; 3 Centro de Informação e Vigilância Sismovulcânica dos Açores (CIVISA), Universidade dos Açores, Rua Mãe de Deus, 9500-801 Ponta Delgada, Azores, Portugal

**Keywords:** Public health, Volcano, Volcanoes, Environmental health

## Abstract

Volcanic CO_2_ diffuse degassing can impact infrastructure, soils, vegetation, microbiota, fauna, and human health. These impacts include acidification of soils, leading to sparse or absent vegetation and changes in microbiota types. Most of the study sites in this review are areas of quiescent volcanism, where soil CO_2_ emissions is a permanent and silent hazard. Lethal indoor and outdoor CO_2_ concentrations measured in different regions of the world (Azores, Aeolian and Canary Islands, Colli Albani, Methana, Massif Central, Mammoth Mountain, Nyiragongo, Nyamulagira, and Rotorua volcanoes) are associated with the asphyxia and death of humans and other fauna (e.g., birds, reptiles, cows, elephants, and dogs). To address the hazard posed by volcanic CO_2_ diffuse degassing, we suggest mitigation measures including mandatory CO_2_ hazard maps for land-use planning, “gas-resistant” construction codes, ventilation mechanisms, monitoring and early warning systems, along with educational campaigns to reduce the gas exposure risks.

## Graphical abstract

**Figure:**
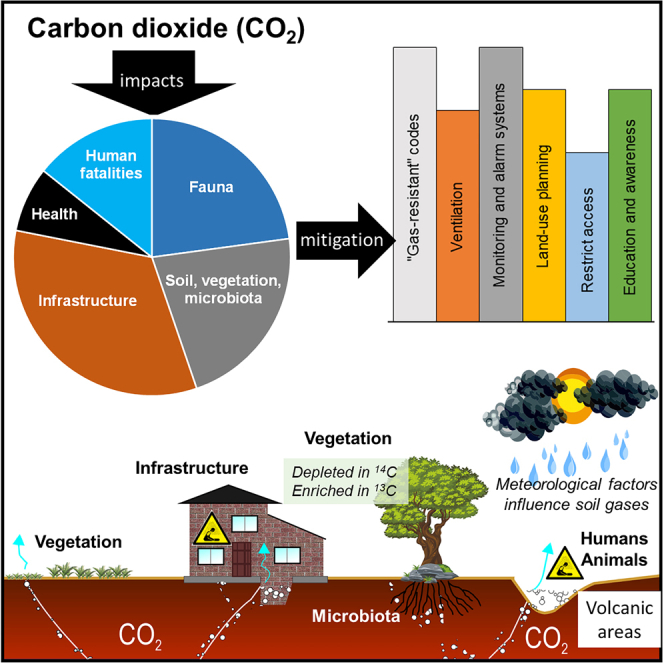

## Introduction

Volcanic gases can pose a permanent threat both during eruptive and quiescent (i.e., the volcano is in a quiet period of activity, but still has potential for future eruptions) periods of activity, as gases may be continuously released from the volcanoes.^1^^,^^2^^,^^3^ Despite visible evidence of gas emissions, such as fumaroles, plumes, and thermal springs, other manifestations are detectable only with specific instrumentation, as is the case of diffuse degassing.^4^^,^^5^ Diffuse degassing areas consist of permanent emission of gases, the predominant one being carbon dioxide (CO_2_), with minor traces of hydrogen sulfide (H_2_S) and radon (^222^Rn). These emissions are usually associated with tectonic structures, such as faults, which act as pathways for the gas ascent.^6^^,^^7^ A study carried out on Mount Etna (Italy) in the early 1990s′ revealed that these invisible and “silent” degassing areas release yearly CO_2_ in the same order of magnitude as the Etna crater’s plumes.^4^

CO_2_, the most abundant volcanic gas after water vapor, is a colorless and odorless gas, which acts as an inert asphyxiant when in high concentrations, and is lethal in concentrations above approximately 100,000 ppm (10 vol. %).^8^^,^^9^^,^^10^^,^^11^^,^^12^ Symptoms associated with high CO_2_ concentrations include headache, shortness of breath, dizziness, nausea, fatigue, and unconsciousness.^12^ This noxious gas is thus a potential permanent hazard not only during volcanic eruptions but also in quiescent, or even, inactive volcanic regions.^2^ At standard temperature and pressure (STP) conditions, CO_2_ is denser than air and can therefore easily accumulate hazardously in poorly ventilated or areas of depression (i.e., caves, holes).^2^^,^^13^ If buildings are placed above diffuse degassing areas, the gas released from soils can eventually ingress into the structure and accumulate, reaching potentially harmful concentrations.^13^^,^^14^^,^^15^^,^^16^

Hansell and Oppenheimer^1^ carried out the first systematic review in 2004 relating health hazards and volcanic gases. The review highlighted the high death toll associated with CO_2_ emissions in the last decades (>2 000 people), referring to some incidents in active and dormant volcanic systems, namely Colli Albani (Italy), Furnas (Azores), Cosigüina (Nicaragua), and Hakkoda (Japan). Most of the reported gas exposure fatalities relate to three incidents, the Dieng Plateau (Indonesia) gas cloud, which caused the death of at least 142 people^9^^,^^17^ and the gas flows released from Cameroonian lakes Monoun (1984) and Nyos (1986). These CO_2_-rich gas clouds caused the death of about 39 and 1700 people at Monoun and Nyos surrounding areas, respectively.^18^^,^^19^^,^^20^ These events relate to non-eruptive CO_2_ emissions and lead in the number of recorded fatalities associated with volcanic gases in the last 600 years.^20^^,^^21^ More recently, Edmonds et al.^2^ highlighted the silent hazards associated with volcanic gases, especially the ones emitted in diffuse degassing areas, and Stewart et al.^22^ carried out the most recent review on volcanic gas impacts. The latter study reviewed the literature published after 2000 to discuss potential health effects associated with volcanic air pollution and showed only two studies,^23^^,^^24^ carried out in the Azores islands, related to diffuse CO_2_ emissions and health impacts. Most of the other publications on this subject were associated with acidic plumes and H_2_S, suggesting an apparent lack of literature on the impacts and hazard assessment in diffuse degassing areas. This review focuses on the impacts that anomalous CO_2_ diffuse degassing may have on the population, infrastructure, and on the environment, accounting for effects on soils, microbiota, vegetation, and fauna. It also presents suggestions for mitigation strategies to reduce the risks associated with diffuse CO_2_ degassing. Recognition of these actions may be particularly relevant considering the recent cases related to the 2021 Vulcano Island unrest (Italy), which resulted in the temporary displacement of populations from some buildings due to gas emissions. Similarly, the hazardous soil CO_2_ emissions identified in Puerto Naos and La Bombilla (La Palma, Canary Islands), and that persisted more than three years after the 2021 Tajogaite volcanic eruption, resulted in inhabitants not being able to return to their homes, highlighting the relevance of mapping and evaluating the potential impacts associated with the silent CO_2_ diffuse degassing zones.

## Results and discussion

Five hundred and sixty-four articles were identified from the five searched databases and 106 were selected for full-text reading after the removal of duplicates and preliminary screening of titles and abstracts (Figure S1). Fifty-eight documents were included in the review after full-text reading the eligible articles (Table S1).

Compared to the previous review,^1^ this study takes into consideration a higher number of articles associated with CO_2_ diffuse degassing. Most of the articles (32) were published in the last 10 years (2014–2024), and only eleven (out of 58) are more than 20 years old. This reveals the increasing interest in diffuse degassing studies in the last two decades and reinforces that, even in periods of quiescence, volcanic hazards need to be accounted for.

### Study sites

Hazardous and impacting diffuse degassing areas were identified in twelve countries, namely in Europe (France, Germany, Greece, Italy, Portugal, Spain), America (USA, Ecuador, Costa Rica), Asia (Japan and New Zealand), and Africa (DR Congo). Most of these emissions occurred in quiescent volcanic systems (Tables 1 and S2). Only seven from the 22 volcanic systems were associated with active volcanoes (Etna, Stromboli, Cumbre Vieja, Turrialba, Irazú, Nyiragongo, and Nyamulagira).^25^^,^^26^^,^^27^^,^^28^^,^^29^^,^^30^^,^^31^^,^^32^^,^^33^^,^^34^^,^^35^

**Table 1:** Main impacts associated to the different study sites (n.r. – not reported)

Study site			Impacts and vulnerabilities				
Country/Region	Volcanic system	State of activity	Buildings or other infrastructure	Environment		Population	
				Soil, vegetation and microbiota	Fauna	Symptoms	Fatalities
Portugal/Azores^13^^,^^14^^,^^24^^,^^36^^,^^37^^,^^38^^,^^39^^,^^40^	Furnas	Quiescent	Indoor air	Plants record^14^C and^13^C imprint of magma-derived CO_2._ Plants are depleted in^14^C and enriched in^13^C. Alteration of lichens exposed to high CO_2_ degassing	Death of animals (birds, cattle, dogs, chickens) in trenches and caves	Persons feeling nausea and vomiting. Respiratory restrictions and COPD (Chronic obstructive pulmonary disease) of exposed individuals	n.r.
Portugal/Azores^41^	Sete Cidades	Quiescent	Indoor air	n.r.	n.r.	Symptoms as headache, dizziness, tiredness, breathing difficulties	n.r.
Portugal/Azores^41^^,^^42^	Fogo	Quiescent	Indoor air	Bared soils, reduced vegetation	Small animals (birds, insects) found dead in depressions and low-ventilated areas	n.r.	n.r.
Italy^43^	Campi Flegrei	Quiescent Unrest	n.r.	^14^C depletion in plants (grass)	n.r.	n.r.	n.r.
Italy^15^^,^^16^^,^^44^^,^^45^^,^^46^^,^^47^^,^^48^^,^^49^^,^^50^^,^^51^^,^^52^^,^^53^^,^^54^	Colli Albani	Quiescent	Indoor air. Gas blowout from shallow boreholes	Lack of vegetation	Hundreds of animals fatalities (cows, toads, sheep, lone foxes, cats, wild pigs, birds, insects, reptiles). Animals die mainly at dawn	Increased risk of mortality (cardiovascular diseases) and increase emergency room visits for diseases of central nervous system	Several reported fatalities. Roberts et al.^47^ report at least 19 fatalities.
Italy^25^	Etna (flank)	Active	Water galleries and boreholes accumulate high CO_2_ concentrations	n.r.	n.r.	n.r.	One fatality reported in a water gallery
Italy^55^^,^^56^	Latera	Inactive (geothermal area)	n.e.	Absence of vegetation. Some acid-tolerant grass resist. Reduced number of plants and fungi in high CO_2_ area. Microbial activity dominated by anaerobic and acidophilic microorganisms	n.e.	n.r.	n.e.
Italy^57^	Pantelleria	Quiescent	Buildings (indoor air)	At CO_2_ concentrations exceeding 180 000–200 000 ppm plants occurred only in small sparsely groups; when concentrations reached 350 000–400 000 or more, vegetation was extremely scarce or even absent	Small dead animals	n.r.	n.r.
Italy^26^	Stromboli	Active	One building exposed to high CO_2_ degassing	n.r.	Dead of small animals close to a mofette (Pizzillo area)	n.r.	n.r.
Italy^58^^,^^59^^,^^60^^,^^61^^,^^62^^,^^63^^,^^64^	Vulcano	Quiescent Unrest	Buildings (indoor air)	Absence of vegetation	Small animals (reptiles, birds) died	Long-term exposure can result in chronic health problems for the occupants of the exposed buildings, who may even be unaware of their houses’ exposure. A child was seriously intoxicated/asphyxiated by the emitted gases	Two children died by asphyxiation in 1998
Germany^65^^,^^66^^,^^67^	East Eifel (Laacher See)	Quiescent	n.r.	Bare soils. Reduced aeration of the soils and increased soil acidification. Grasses are more tolerant to high soil CO_2_. Microbial shift toward anaerobic and acidotolerant to acidophilic species. Decrease on plant coverage and diversity in areas with high soil CO_2_	n.r.	n.r.	n.r.
Greece^68^	Methana	Quiescent	n.r.	n.r.	Dead of small animals (insects, small rodents and reptiles) in the vicinities of a hole that is about 1.5 m deep and 1 m in diameter	n.r.	n.r.
France^69^	Massif Central	Quiescent	Buildings (indoor air)	n.r.	n.r.	n.r.	n.r.
Spain/Canary Islands^27^^,^^28^^,^^29^	Cumbre Vieja	Active	Buildings (indoor air)	Agricultural fields affected. Banana plantation with approximately 4 200 m^2^ affected	Dead animals found in the area	n.r.	n.r.
Costa Rica^30^^,^^31^	Irazú and Turrialba	Active	n.r.	Increase in vegetation kill zone area (2007–2008) should be at least partly due to soil CO_2_ diffuse degassing. Incorporation of volcanic CO_2_ into the biomass. Functional response of some species (increase stomatal conductance and chlorophyll concentration)	n.r.	n.r.	n.r.
Ecuador^70^	Cuicocha	Quiescent	n.r.	Dead vegetation	n.r.	n.r.	Death of 6 persons by CO_2_ in a thermal-spa (5 km south of Cuicocha Volcano)
USA^71^^,^^72^^,^^73^^,^^74^^,^^75^	Mammoth Mountain	Quiescent	n.e.	Tree-kill areas (∼50 ha in the summer 1995)	Rodents and birds found death	Severe symptoms of asphyxia	A skier died in a depression with about 700 000 ppm of CO_2_ with acute pulmonary edema
DR Congo/Virunga area^32^^,^^33^^,^^34^^,^^35^	Nyiragongo and Nyamulagira	Active	Swelling of the pavement in buildings from Goma due to CO_2_ accumulation	Specific vegetation depending on the CO_2_ concentration. For CO_2_ > 500 000 ppm vegetation does not develop (bare and weathered rocks; acidified soils)	Dead animals (insects, birds, lizards, digs, rats or snakes) are common. Dead elephants, lions, hippos, buffalo, hyenas, monkeys, cows, goats have also been found	Severe symptoms of asphyxia	Number of deaths not available, however should occur every year. For example, 37 deaths in 2007
Japan^76^	Hakkoda	Quiescent	n.r.	n.r.	Death animals	n.r.	3 deaths by asphyxia in a topographic depression
New Zealand^77^	Rotorua	Quiescent	Buildings (indoor air)	n.r.	Dead animals outdoor (birds) and indoor (cockroaches)	n.r.	n.r.

Ref. respects to the references. ∗ corresponds to the abstracts manually included.

### Soil CO_2_

**Figure 1:**
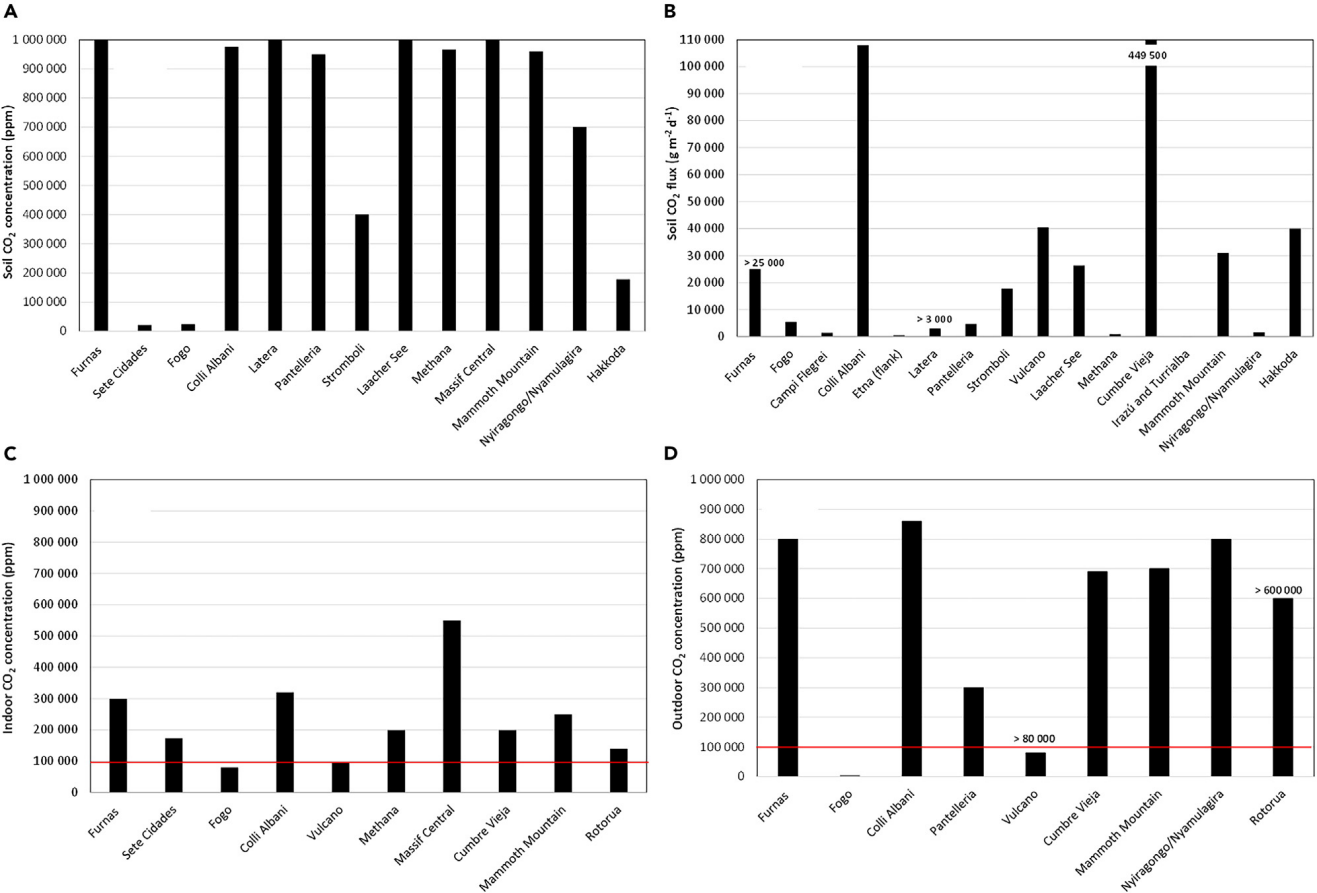
Maximum soil CO_2_ concentrations (A) and fluxes (B) measured at the different sites according to the included studies. (C and D) refer, respectively, to indoor and outdoor CO_2_ concentrations. Red lines represent lethal concentration (∼100 000 ppm) for CO_2_ exposure. Some studies do not show the exact value of CO_2_ concentrations or fluxes, and in these cases the graphic displays the maximum values mentioned (numbers with “>”).

CO_2_ concentrations were measured in the soil air at different depths (from about 20 cm to ∼1 m depth), depending on the study (Figure 1). The highest soil CO_2_ concentrations reached 100 vol. % (1 000 000 ppm) at several sites, meaning that the entire soil air is filled with CO_2_. This happened at the quiescent, or inactive, volcanic sites of Furnas, Latera, Lacher See and Massif Central.^36^^,^^55^^,^^65^^,^^69^ Hernández et al.^27^ determined the highest CO_2_ fluxes (449 500 g m^−2^ d^−1^) at La Bombilla (Cumbre Vieja). Carapezza et al.^45^ measured a similar order of magnitude of fluxes at Colli Albani. Soil gases showed clear deep-derived contribution for the CO_2_ concentrations and fluxes, well above the biogenic values related to the soil respiration (usually for concentrations and fluxes up to 50 000 ppm and 50 g m^−2^ d^−1^, respectively).^78^

### Outdoor CO_2_

Outdoor CO_2_ concentrations were measured at different heights (from the ground and up to 130 cm height) and in cavities or topographic depressions. In this review, we report the highest CO_2_ concentrations measured in each study, with the intent to show the most hazardous levels. Lethal outdoor CO_2_ concentrations (>100 000 ppm) were measured at eight sites, namely at Furnas,^36^^,^^37^ Colli Albani,^15^^,^^48^ Vulcano,^59^ Pantelleria,^57^ Cumbre Vieja,^28^^,^^29^ Mammoth Mountain,^73^ Rotorua,^77^ Nyiragongo, and Nyamulagira^33^ (Figure 1). These measurements are in agreement with registered impacts, since in most of the above-referred sites, excluding Pantelleria and Rotorua, deaths and serious symptoms were reported as affecting the population.

### Indoor CO_2_

With regards to indoor CO_2_ concentrations, lethal values were also measured at Furnas,^13^^,^^14^^,^^36^ Sete Cidades,^41^ Colli Albani and Mts. Sabatini,^15^^,^^46^^,^^49^ Vulcano,^59^ Mammoth Mountain,^73^ Methana,^68^ Cumbre Vieja,^28^ Massif Central,^69^ and Rotorua^77^ (Figure 1). No indoor CO_2_ concentrations for Nyiragongo and Nyamulagira were found in the literature, even if lethal concentrations are expected considering the soil/outdoor concentrations, as well as the symptoms and deaths reported.

The highest indoor CO_2_ concentrations were measured in basements and at ground floor levels, which are associated not only with the proximity to the gas source (soil) but also with the lack of natural ventilation.

### Impacts

**Figure 2:**
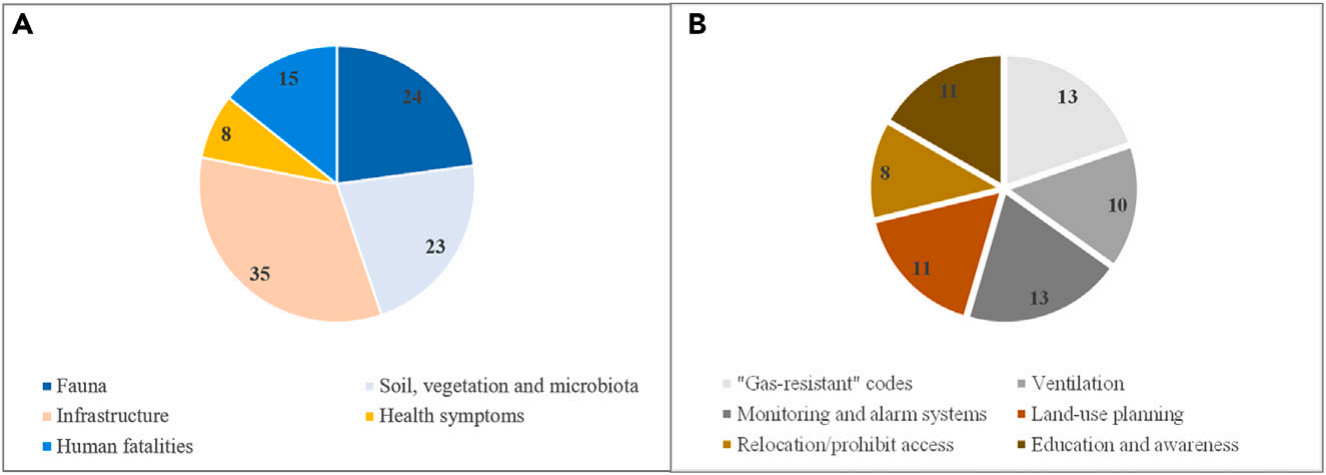
Number of studies included in the review that report (A) different CO_2_ degassing impacts, and (B) mitigation strategies.

Several volcanic areas are affected by hazardous CO_2_ diffuse degassing that may impact not only human health and infrastructure,^13^^,^^14^^,^^15^^,^^16^^,^^24^^,^^36^^,^^37^^,^^38^^,^^39^^,^^40^^,^^41^^,^^42^^,^^25^^,^^44^^,^^45^^,^^46^^,^^47^^,^^48^^,^^49^^,^^50^^,^^51^^,^^52^^,^^53^^,^^54^^,^^57^^,^^64^^,^^27^^,^^28^^,^^29^^,^^69^^,^^32^^,^^33^^,^^34^^,^^35^^,^^77^ but also the environment with areas of altered soils,^66^^,^^67^ absent vegetation,^38^^,^^42^^,^^55^^,^^57^^,^^59^^,^^65^^,^^30^^,^^71^^,^^72^^,^^73^ and animal casualties varying from insects to large animals, such as cows and elephants^37^^,^^44^^,^^45^^,^^68^^,^^74^^,^^32^^,^^33^ (Table 1; Figure 2A).

### Human impacts

Health symptoms in the population due to high CO_2_ concentrations include nausea, vomiting, headache, dizziness, tiredness, increased breathing rate, and loss of consciousness^13^^,^^41^ (Table 1). The reported symptoms are usually associated with reduced oxygenation and are essentially caused by acute exposure to high CO_2_ emissions. CO_2_ acts as an asphyxiant, and the lethal threshold for humans and animals is above 100 000 ppm. Above 200 000 ppm, CO_2_ can lead to sudden loss of consciousness and death from acute hypoxia, severe acidosis, and respiratory paralysis after only a few breaths.^75^

Even given these serious health effects, epidemiological studies related to CO_2_ poisoning in degassing areas are almost absent in the reviewed literature. Respiratory restrictions and COPD were associated with higher CO_2_ exposure at Furnas Volcano,^24^ and recently, Carapezza et al.^16^ reported an increased risk of mortality due to cardiovascular diseases or central nervous system issues at Colli Albani (Italy). Epidemiological studies of the effects of long-term (chronic) exposure to anomalous CO_2_ concentrations are thus missing. This review reports several areas (e.g., Azores, Canary and Aeolian Islands, Massif Central, Cuicocha, Nyiragongo and Nyamulagira volcanoes) which could be used as test sites for future studies.

Fifteen studies reported fatal incidents with at least 73 fatalities in Italy (Colli Albani, Etna and Vulcano),^47^^,^^25^^,^^62^ Ecuador (Cuicocha),^65^ USA (Mammoth Mountain),^74^ Japan (Hakkoda),^76^ Nyiragongo and Nyamulagira (DR Congo).^32^^,^^33^ In the outdoor cold CO_2_ emissions, referred as *mazuku* by the local population^33^ in the Democratic Republic of Congo, people die every year, although there are no official numbers available.^32^^,^^33^^,^^34^ Smets et al.^32^ report the death of 37 individuals in 2007 associated with the *mazuku*, and these deaths are probably missing from the count of volcanic fatalities.^20^^,^^21^ This review article thus suggests that the estimated human death toll associated with soil CO_2_ diffuse degassing sites^1^^,^^2^^,^^21^ is well below the real numbers.

### Impacts on animals

Twenty-four articles report the death of several animals, from insects and rodents to large animals, such as elephants, lions, hippos, cows, and buffalos.^32^^,^^33^ No articles identify specific symptoms on the fauna or even report CO_2_ concentrations associated with the deaths. As far as we are aware, these studies have not been conducted as they are not available in the literature. The animals are usually found dead in the degassing areas.

### Environmental impacts

Twenty-three studies mention the impact of high CO_2_ levels on the vegetation, and, even if Pfanz et al.,^65^ and references therein, reported that some plants are mofettophilic, i.e., well adapted to the presence of anomalous high CO_2_ in the soil, most of the species do not develop well, or may even die, in the presence of high soil CO_2_ emissions. Acidified and less aerated soils^66^ are probably the main causes for the lack of vegetation identified in several diffuse degassing areas, such as in the case of Mammoth Mountain (USA),^72^ where about 50 ha of vegetated area was destroyed in the 1980’s- 1990’s due to maximum soil CO_2_ concentrations and fluxes of 900 000 ppm and 31 000 g m^−2^ d^−1^, respectively.^72^ As mentioned, CO_2_ degassing decreases the soil’s pH as well as the redox potential, impacting also the microbial diversity, which is usually reduced and dominated by anaerobic and acidophilic microorganisms.^55^^,^^56^^,^^66^^,^^67^

**Figure 3:**
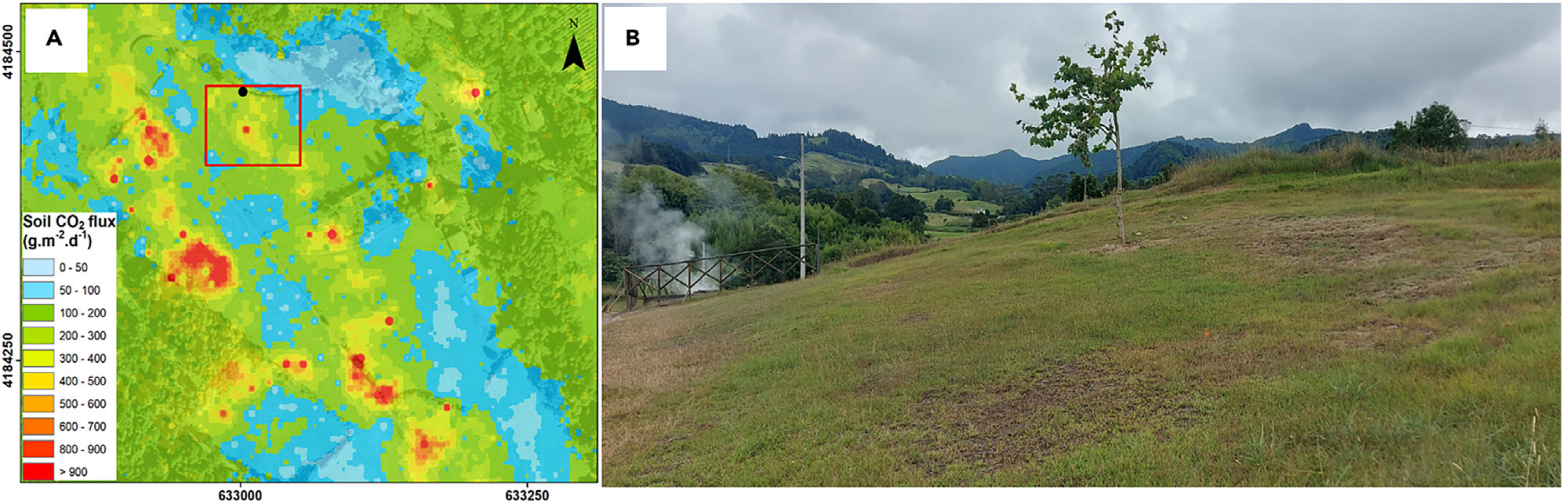
Colored map (A) showing the amount of CO_2_ emitted from the soils in a degassing area of Fogo Volcano (Azores archipelago). The red circle corresponds to the picture (B), where the impact of anomalous CO_2_ degassing and temperature on the vegetation is visible. The brownish vegetation corresponds to the areas where the emission of CO_2_ is higher. Soil CO_2_ flux map modified from Viveiros et al.^42^

D’Alessandro et al.^57^ reported that vegetation is very scarce in Pantelleria when soil CO_2_ concentrations reach ∼350 000 ppm, which is in line with an observation from Smets et al.^32^ for Nyiragongo and Nyamulagira, where vegetation does not develop where there is more than 500 000 ppm of CO_2_ in the soil. These thresholds change depending on the study site, and Frerischs et al.^66^ defined concentrations in the soil between 100 000 and 200 000 ppm as the levels responsible for creating anaerobic habitats and decreasing the microbial activity in the soils. The remote detection of the effect of CO_2_ on vegetation is the basis for a recent study^79^ that argues that plants can be sensitive to minor changes in volcanic activity. This is in agreement with a study carried out in the Azores,^80^ which shows that the impact on the vegetation is even more significant if there is a soil thermal anomaly (Figure 3).

Bare and weathered soils^66^ may affect agriculture and, consequently, the local economy. Following the 2021 La Palma eruption, CO_2_ also reached high outdoor concentrations (∼690 000 ppm) in a banana plantation in the area of La Bombilla,^29^ causing the death of several animals and consequent social and economic impacts.

Impacts of CO_2_ on biomass may also lead to the functional response of some species with increased stomatal conductance and chlorophyll concentration.^31^ Other studies at Furnas^39^ and Campi Flegrei^43^ also showed different carbon imprints on plants located in diffuse degassing areas. In these cases, plants exposed to volcanic degassing are depleted in ^14^C since they assimilate from soil CO_2_ free of ^14^C. This recalls the possibility of biased ^14^C dating of volcanic deposits, as organic samples dated with this methodology result in apparent higher ages.^43^ In other cases, this has been seen as an opportunity, and it was suggested that the ^14^C analysis of selected plants in active volcanic areas could provide an opportunity to quantify local CO_2_ fluxes.^43^

### Impacts on buildings and infrastructure

High temperature and acidic volcanic gases^81^ can cause damage to the structure of buildings, leading, for example, to metallic corrosion. Corrosion is not commonly associated with anomalous cold CO_2_ emission, however, when buildings are located above diffuse degassing areas, gas can ingress into the building and reach lethal concentrations. Several articles (29) report hazardous indoor CO_2_ exposure related symptoms, which can be associated with the “sick building syndrome” (SBS).^82^ This term refers to situations in which building occupants experience acute health and comfort effects (e.g., headache, throat irritation, coughing or sneezing, fatigue, difficulty in concentration) that seem to be linked directly to the time spent in the building. For the cases where access to these buildings may be restricted or completely barred, volcanic gases are considered to have an impact on infrastructure (Figure 2A).

Lethal indoor CO_2_ concentrations have been detected in the Azores islands,^13^^,^^14^^,^^41^ Colli Albani,^15^^,^^16^^,^^46^^,^^50^ Vulcano,^59^ Cumbre Vieja,^27^^,^^28^ Mammoth Mountain^73^ or Rotorua.^77^ CO_2_ emitted from the soil beneath the buildings is the main cause of indoor CO_2_, rather than the influx of gases from outdoors.^64^ In some cases, buildings become non-habitable resulting in the displacement of their inhabitants.^14^^,^^16^^,^^41^ Due to the odorless and colorless characteristics of CO_2_, there is the possibility that inhabitants are not aware of their home’s exposure to CO_2_ emissions, as suggested by Granieri et al.^60^ at Vulcano Island.

Nevertheless, accessing the buildings and performing indoor measurements can be quite challenging, as only four of the 58 studies report indoor CO_2_ time series. Challenges include difficulties in getting authorization from the owners to access buildings and perform measurements, limiting the current knowledge on the potential impact diffuse degassing areas have on the population. Mistrust of the scientific community as well as anxiety about hazardous indoor concentrations being detected (which might result in economic loss and inhabitants’ relocation) are some of the factors that make access difficult. Residents also need to be reassured that they will be informed about the results and that confidentiality will be guaranteed.^77^ Nevertheless, ethical questions arise when hazardous concentrations are measured indoor, since researchers have the responsibility to inform civil protection authorities in the cases where hazardous concentrations are detected, meaning that this needs to be clarified and agreed upon with residents before the research is undertaken.^77^ Real-time monitoring systems, such as the recent installations at Vulcano^64^ and La Palma,^28^ will result in a significant increase in data availability, which in turn will contribute to a better understanding of indoor concentrations and the correlation between soil gases and indoor/outdoor CO_2_ variations.

Structural impacts were reported in the literature for buildings including the swelling of the pavement in buildings at Colli Albani^16^ and Goma (DR Congo) due to the pressure caused by the CO_2_ accumulation.^33^ Partial collapses of some roads in the Colli Albani area^16^ with some gas released were also associated with diffuse degassing and constitute a type of infrastructure impact, which was not reported elsewhere.

Other affected infrastructure includes boreholes drilled in diffuse degassing areas where shallow gas-pressurized aquifers are present, which can result in hazardous gas blowouts. This situation has been reported only in the Colli Albani area (Italy).^16^^,^^46^^,^^50^^,^^51^^,^^52^^,^^53^ The sudden emission of gases (essentially CO_2_ and H_2_S) may be lethal for animals,^51^^,^^52^^,^^53^ and, in some cases, may create soil gas anomalies and reach residential areas, also causing high indoor gas concentrations.^53^

### Hazard maps

Even though several studies have been carried out to evaluate hazardous gas concentrations in diffuse degassing areas, the literature still lacks hazard maps to assess volcanic CO_2_ diffuse emissions, especially concerning indoor concentrations. We consider these maps as mandatory tools in degassing areas for both local civil protection and land-use planners, as soil gas levels should be known before the authorization of any construction and/or to assist in crises management decision-making process. Hazard maps should couple soil degassing (concentrations and/or fluxes), carbon isotopic data, and air CO_2_ quantifications (indoor and outdoor) to define criteria that could be applied worldwide. Pareschi et al.^52^ proposed simulations to assess outdoor CO_2_ concentrations at Vulcano Island based on soil CO_2_ fluxes, gas output from crater fumaroles, topography, and weather conditions. This type of approach is comparable to the dispersion models applied more recently by Granieri et al.^60^ or Viveiros et al.,^42^ respectively, at Vulcano and Caldeiras da Ribeira Grande. The resulting simulations resemble outdoor hazard maps. The dispersion models already being implemented^60^^,^^83^ need to be improved to account also for the existence of thermal anomalies, and the potential interference of other hazardous gases.

Another approach to produce gas hazard maps has been proposed for the area of Lavinio-Tor Caldara at the periphery of Colli Albani.^84^ In this case, sites with gas discharges, pressurized aquifers, the occurrence of previous incidents, high soil CO_2_ concentrations (>400 000 ppm at 50 cm depth), and CO_2_ fluxes (in the case, >65 g m^−2^ d^−1^) are considered as hazardous. The potential gas hazard-prone area is defined as the wrapping of circles with a radius of 0.5 km centered on each hazardous site.

For the indoor hazard maps, only two studies^17^^,^^41^ suggested soil gas levels as criteria to produce hazard maps, and, even if carried out in different areas (Azores and Colli Albani), the levels identified were quite similar. Difficulties to access indoor data (as discussed by Gal et al.^59^) are likely one of the main limitations for developing complete and integrated soil-outdoor-indoor maps.

Barberi et al.^15^ argue that any building located in areas with soil CO_2_ above 10 000 ppm should already require structural actions, and soils with CO_2_ above 50 000 ppm should be classified as “no building areas”. These levels are in agreement with the ones defined by Viveiros et al.^38^ that considered high risk of asphyxia for areas with soil CO_2_ concentration above 50 000 ppm. These defined thresholds need to take into consideration the potential effect meteorological factors may have on gas variations, since lethal concentrations may be reached only due to extreme weather conditions.^14^

Only one article in the review suggests criteria to produce CO_2_ exposure risk maps,^40^ and integrates hazard, exposure, and vulnerability of the buildings, highlighting the urgent need to develop studies that would support land-use planners.

One aspect that needs to be taken into consideration is that hazard maps are dynamic and unrest episodes, and/or volcanic activity may change the hazard defined for an area, as recently observed at Vulcano^63^ or La Palma^27^ islands.

### Factors interfering with CO_2_ concentrations

Outdoor and indoor CO_2_ concentrations time series recorded in the different studies show significant short-term^65^ and long-term variations,^13^ which can be justified by several causes, including volcanic and seismic activity, as well as the interference of environmental variables.^13^^,^^14^ The influence of all these factors is crucial to understanding how gas fluxes may change and, consequently, creating the need for dynamic hazard maps, which should be updated regularly.

#### Variations associated with volcanic activity

In terms of volcanic influences on CO_2_ concentrations, the ongoing 2021 unrest episode at Vulcano Island increased gas emissions in the crater and in the flanks through diffuse degassing. This resulted in the temporary relocation of the residents of some of the buildings, only allowing them access their homes during daytime.^63^^,^^64^

The CO_2_ degassing zones that developed at Puerto Naos and La Bombilla after the 2021 Tajogaite volcanic eruption (La Palma) constitute another example of how volcanic activity may increase significantly the hazards, causing local inhabitants to be impeded from returning to their homes.

To the best of our knowledge, these lethal CO_2_ concentrations measured after the end of the eruption, about 5 km away from the eruptive vents, was the first case ever monitored. However, this situation reminds us of historical accounts associated with the 1808 volcanic eruption in São Jorge Island (Azores) that reported the simultaneous death of three persons in a tide well two years after the end of the eruption, a few kilometers away from the vents.^85^ We believe that these historical accounts are a proxy of what is now observed at La Bombilla and Puerto Naos (La Palma), where hazardous and lethal CO_2_ concentrations are still measured hampering residents to return to their apparently intact homes. Seismicity may likewise increase permeability and new degassing areas can emerge.

#### Anthropogenic activities

The drilling of a geothermal well close to an inhabited area in the Azores archipelago resulted in the expansion of a diffuse degassing area, showing that anthropogenic activities can also potentiate hazardous CO_2_ levels.^42^^,^^80^ Cementing some streets, for instance, may also interfere with the degassing path, and gas that was freely released from the soil finds other paths, possibly traveling to inhabited areas. This type of situation occurred at Mosteiros village in the Azores.^41^

#### Meteorological influences

As mentioned before, CO_2_ diffuse degassing may be affected not only by deep processes, such as volcanic and seismic activities but also by meteorological/environmental factors, which have been shown to interfere significantly with the gas emissions. For example, statistical approaches applied to indoor CO_2_ data showed that about 30% of the CO_2_ variability was due to changes in barometric pressure, wind speed, and soil water content.^13^ Several studies^13^^,^^14^^,^^41^ showed that indoor gas increases to lethal concentrations only due to fluctuations of a few mbar in the atmospheric pressure. Carapezza et al.^16^ recently showed significant changes in the indoor CO_2_ and H_2_S time series recorded in buildings at Cava dei Selci (Colli Albani), with lower concentrations registered during the winter months compared to the spring-summer period. These lower concentrations during winter were explained by the heavy rainfalls that enriched the aquifers with meteoric water, dissolving more gases when compared with the spring-summer time. Consequently, a decrease in gas released into the atmosphere is observed. Camarda et al.^61^ did not display indoor CO_2_ concentrations but discussed that soil CO_2_ flux increases, up to three orders of magnitude, due to variations in barometric pressure, particularly in sites with low soil gas fluxes.

One additional aspect that is worth discussing is related to the seasonal trends recognized in the soil CO_2_ flux time series.^13^ Most of the studies carried out up to present day in the Azores archipelago revealed that indoor CO_2_ is higher during the winter season compared to the summer period.^13^^,^^14^^,^^17^ This seasonality was explained as resulting from the coupled effect of the barometric pressure and the reduced natural ventilation during extreme weather conditions. Viveiros et al.^13^^,^^14^ also discussed the effect of rainfall, as during the rainy periods the soil voids are saturated with water and do not allow an easy escape of the gas through the surface. In these conditions, gas travels more easily below the buildings, where the soil is still dry, and if it finds a pathway it can accumulate inside buildings increasing indoor concentrations.

Weather conditions also interfere significantly with outdoor gas concentrations, especially the wind speed that can dilute the gases when it is high, or contrarily allow their accumulation.^45^^,^^48^ Together with the effect of the wind, insolation is also a parameter that can control the accumulation of gas, since with the warming-up of the surface the accumulated gas tends to rise. Contrarily, during the night, and in the absence of solar radiation and lower wind circulation, cold CO_2_ accumulates closer to the soil’s surface,^57^^,^^77^^,^^84^ as it is denser than air (at STP conditions).^75^ Carapezza et al.^45^ reported the death of animals in the Colli Albani area mainly at dawn, which agrees with the wind-insolation effects discussed above. This behavior was similarly described at the *mazuku*, also called "evil winds that travel and kill during the night”,^32^ recalling the fact that meteorological factors highly interfere with the gas dispersion causing CO_2_ concentrations to increase to lethal levels during the night in these sites.^32^^,^^33^

The included studies however did not address the potential effects of thermal anomalies, which are often associated with CO_2_ emissions. When the soil temperature is anomalous, CO_2_ density changes and gas may rise higher away from the ground instead of accumulating nearer to it. For this reason, land-use planners should also consider soil temperature mapping carried out on the degassing areas.

### Correlation with other gases

**Figure 4:**
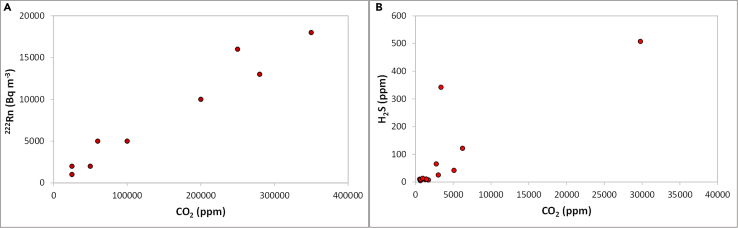
Relation between indoor CO_2_ and other volcanic gases (A) Relation between indoor radon and CO_2_ at Furnas Village (Azores archipelago) (based on data from Baxter et al.^36^), and (B) H_2_S and CO_2_ in buildings at Colli Albani (based on Carapezza et al.^48^).

CO_2_ can be associated with other gases, such as the hydrogen sulphide (H_2_S)^48^ or the radioactive radon (^222^Rn)^36^ (Figure 4). High indoor radon concentrations were measured coupled with CO_2_ at Colli Albani^44^ and Furnas^36^ volcanoes, suggesting that CO_2_ may act as carrier gas. This study did not aim to focus on the impact of other gases, however, the coupled effect of CO_2_-^222^Rn on population health should be studied, as radon is recognized as the second highest cause of lung cancer,^86^ with the few studies focusing on CO_2_ health impact also showing correlation with respiratory and cardiovascular diseases.^16^^,^^24^

H_2_S is a toxic asphyxiant gas^11^ and several studies^15^^,^^16^^,^^48^^,^^49^^,^^59^^,^^62^ showed its presence associated with high CO_2_ emissions. Instantly lethal concentrations (>1 000 ppm) were measured at Colli Albani, suggesting that this gas is the most hazardous volatile in this volcanic area.^15^^,^^48^^,^^49^^,^^84^ Hazardous H_2_S concentrations (>80 ppm) were also measured at Vulcano.^62^

### Mitigation actions

Most of the included studies (35) recommended mitigation strategies to address the impact of anomalous high CO_2_ emissions. Table 2 details all the mitigation actions recommended on the selected studies. Strategies were classified into six categories and include mostly what could be denominated “gas-resistant” codes,^13^^,^^14^^,^^40^^,^^41^^,^^44^^,^^46^^,^^51^^,^^52^^,^^53^^,^^69^^,^^32^^,^^34^^,^^77^ implementation of ventilation^13^^,^^14^^,^^15^^,^^24^^,^^36^^,^^40^^,^^45^^,^^46^^,^^34^^,^^77^ and real-time monitoring systems,^13^^,^^14^^,^^15^^,^^24^^,^^36^^,^^40^^,^^49^^,^^53^^,^^62^^,^^63^^,^^64^^,^^70^^,^^73^ production of hazard maps that should be used by land-use planners,^15^^,^^37^^,^^40^^,^^42^^,^^44^^,^^45^^,^^54^^,^^58^^,^^61^^,^^73^^,^^32^ as well as more long-lasting actions, such as the relocation of inhabitants and restrict access to the dangerous areas^16^^,^^45^^,^^46^^,^^47^^,^^25^^,^^64^^,^^73^^,^^35^ (Figure 2B). The importance of education and distribution of informative pamphlets were also considered by several studies,^13^^,^^15^^,^^16^^,^^24^^,^^45^^,^^59^^,^^69^^,^^32^^,^^33^^,^^35^^,^^77^ highlighting that together with technical solutions, human behavior needs to be addressed to reduce the risk.

**Table 2:** Recommended mitigation strategies compiled based on the reviewed literature

Recommended mitigation strategies	References
**“Gas-resistant” codes** • Gas-proof (impermeable) membranes • Ventilated subfloor void (raised floors) • Sealing gaps in the foundations, fill crack with sand and cement • Introduce siphon drains (pipes) • Cementation of the boreholes (use also BOP when drilling) • Avoid/prohibit basements	Viveiros et al.^13^, Viveiros et al.^14^, Viveiros et al.^40^, Viveiros et al.^41^, Annunziatellis et al.^44^, Barberi et al.^46^, Carapezza et al.^51^, Carapezza et al.^52^, Carapezza et al.^53^, Frédérick et al.^69^, Smets et al.^32^, Boudoire et al.^34^, Durand and Scott^77^
**Ventilation systems (natural and/or forced)** • Natural ventilation in all compartments • Ventilation systems for basement and ground floor rooms • Installation of positive-pressure air conditioning	Viveiros et al.^13^, Viveiros et al.^14^, Barberi et al.^15^, Linhares et al.^24^, et al.^36^, Viveiros et al.^40^, Carapezza et al.^45^, Barberi et al.^46^, Boudoire et al.^34^, Durand and Scott^77^
**Monitoring and alarm systems** • Monitoring and alarm systems installed in public buildings • Installation of indoor air CO_2_ (and H_2_S) monitoring network with automatic alert system and forced air ventilation systems • Synchronous measurements of soil CO_2_ fluxes and air CO_2_ concentrations to establish a warning system for gas hazards • Meteorological forecasts as proxy of indoor CO_2_ increases	Viveiros et al.^13^, Viveiros et al.^14^, Barberi et al.^15^, Linhares et al.^24^, et al.^36^, Viveiros et al.^40^, Roberts et al.^49^, Carapezza et al.^53^, Diliberto et al.^62^, Di Martino et al.^63^, Gurrieri et al.^64^, Sierra et al.^70^, Farrar et al.^73^
**Land-use planning** • Map soil gas areas (soil gas and/or gas flux measurements) • Couple CO_2_ distribution maps and gas dispersion models • Carbon isotopic data to identify gas sources and should be used to elaborate hazard maps • Understanding the influence of environmental parameters is crucial to develop degassing hazard maps	Barberi et al.^15^, Viveiros et al.^37^, Viveiros et al.^42^, Viveiros et al.^40^, Annunziatellis et al.^44^, Carapezza et al.^45^, et al.^54^, Pareschi et al.^58^, Camarda et al.^61^, Farrar et al.^73^, Smets et al.^32^
**Relocation/Prohibition of access to dangerous sites** • Displacement of people from buildings • Relocation of population during increase volcanic outgassing • Avoid regions of low topography • Avoid activities with breathing at ground surface • Close of a campground in the tree-kill area • Warning signs and close accesses to anomalous areas • Delimit the hazardous places for land-use planning	Carapezza et al.^16^, Carapezza et al.^45^, Barberi et al.^46^, Roberts et al.^47^, D’Alessandro^25^, Gurrieri et al.^64^, Farrar et al.^73^, Macumu et al.^35^
**Educational and awareness campaigns** • Informative booklets with precautionary measures • Periodic campaigns to increase risk awareness of the residents • Preventive campaigns and signs in the *mazuku* sites • Promote informative actions about “good practices” on how to face gas hazards • During constructions: inform workers and use safety gas detectors • Follow-up health programs in order to provide medical counselling (when applicable)	Viveiros et al.^13^, Barberi et al.^15^, Carapezza et al.^16^, Linhares et al.^24^, Carapezza et al.^45^, Carapezza et al.^59^, Frédérick et al.^69^, Smets et al.^32^, Balagizi et al.^33^, Macumu et al.^35^, Durand and Scott^77^

Mitigation actions can be divided in two steps: before construction and after buildings are found in a degassing area. Any construction above a low to moderate risk diffuse degassing area should follow specific construction rules to reduce the risk, since even recent and modern constructions can be vulnerable to gas ingress. The denominated “gas-resistant” codes^13^ should be legislated, comprising prohibiting basements or any underground structure, installing impermeable membranes between soil and ground (whenever possible), and guaranteeing natural ventilation in all compartments. Another possibility that architects and engineers should implement is the creation of a vented space between the soil and the ground floor (suspended floor) which would potentiate the aeration of soil gases before their ingress into the building. Construction of buildings should not be authorized in areas classified as high CO_2_ risk. When the degassing anomaly is recognized after buildings are in use, the remedial actions should be focused on the building’s structure, with the implementation of impermeable membranes and setting up of ventilation systems together with monitoring and early warning systems. The interference of meteorological factors on the CO_2_ emissions that increase indoor concentration during winter periods reveals the need to implement artificial ventilation systems, since the natural ones may not be efficient during bad weather conditions.

We believe that any public building related to health care services and vulnerable populations (i.e., schools, nursing homes) located in diffuse degassing zones, even if classified as low to moderate risk of exposure, should be regularly monitored by means of a real-time system. Caves, and any other underground structure located in diffuse degassing areas, should implement a real-time monitoring system to inform and control visitors’ or workers’ access to the area.

Few studies that continuously measure CO_2_ concentrations associated with early warning systems are available; however, the ongoing hazardous situations of Vulcano and La Palma islands potentiate the development of low-cost gas sensors that can be implemented as early warning systems, and are crucial to monitor a large number of buildings. We suggest that indoor monitoring systems may need to be coupled with automatic ventilation systems and that networks should also account for sensors on the upper floors, especially if buildings are located over a thermally anomalous area. Regular planned sensor calibrations, as well as information on potential maintenance of the system, are some of the aspects that need to be accounted for when defining these monitoring systems.

Installation of monitoring and early warning systems is successful only if residents are trained to understand the recorded values and the alert levels, as well as to follow the instructions arising from the different defined levels. Risk communication needs to be implemented through informative sessions. We suggest prioritizing the individual approach, when possible, to assure the understanding of the instructions. Despite all the mitigation actions, in some cases of high risk of exposure, the only strategy is relocation, evacuation, restricting or forbidding access to the hazardous sites. Identification of CO_2_ anomalous degassing zones is crucial for locals and tourists. Several of the identified degassing areas are also quite attractive for tourists, who in some cases decide to do nature-based activities, including camping, lying on or near the ground surface, or even walk in some not so well-ventilated trails. Due to the potential accumulation of lethal CO_2_ also outdoors, in depressions or low-ventilated areas, appropriate signage in these sites should be mandatory.

This study accounts for soil CO_2_ degassing zones associated essentially with active and quiescent volcanoes. Potential diffuse CO_2_ from non-volcanic tectonic structures were not included. In addition, impacts caused by the release of CO_2_-rich clouds from volcanic lakes (e.g., lakes Monoun and Nyos) were excluded from the analyses. In the case of volcanic lakes, even if the source of gas is the same, the mechanisms that cause the incident and the remediation strategies are significantly different.

## Conclusions

Diffuse degassing studies have been mostly developed in the last 30 years, and due to the high CO_2_ concentrations in the atmosphere, studies in these areas are essentially carried out through direct measurements. Due to the difficulty to access some areas and/or the time-consuming process associated with field surveys, several diffuse degassing areas worldwide may still be unidentified. This review highlights that even areas without volcanic eruptions in the last centuries and where the probability of occurring an eruptive event is low, may still be affected by hazardous CO_2_ diffuse degassing. Massif Central (France) and Eiffel (Germany) are two of these cases.

Very few hazard maps are available in the literature. Epidemiological studies able to evaluate the impact of long-term (chronic) exposure in areas with permanent soil CO_2_ diffuse degassing are also lacking, and the fatalities reported in the literature related to acute exposure to CO_2_ are probably underestimated. In urbanized areas, CO_2_ entering in buildings can reach lethal concentrations and cause symptoms for the residents. These buildings may become unsuitable for living, and be classified as “sick buildings”. Structural effects associated with CO_2_ degassing in buildings are very few and comprise essentially of swelling of floors due to the pressure of soil gases. Gas blowouts resulting from boreholes and partial collapse of roads are other infrastructure impacts recognized in the literature.

Bare and acidified soils due to the CO_2_ emission decrease vegetation coverage and diversity. Anaerobic and acidophilic microorganism also dominate microbial activity existing in the soil. If on one side this impacts the environment and interferes with the local economy due to the impact on cultivated lands,^42^^,^^83^ on the other it can be seen as an opportunity to remotely monitor volcanic systems.^80^ CO_2_ released from soils is also captured by the plants and may result in biased data when dating volcanic rocks with ^14^C, increasing the potential ages of the studied products.

Considering this “silent” hazard, further studies evaluating the perception of the population would be welcome. When speaking about education, and considering the lack of epidemiological studies carried out in diffuse degassing areas, we feel that sessions to target audiences, such as healthcare professionals, authorities, land-use planners, civil engineers, and architects, will contribute to facing the impacts of these silent and often unknown sites. Implementation of multidisciplinary studies within the scientific community (including psychologists, health professionals, biologists, risk communicators, architects, and civil engineers) is mandatory to understand diffuse degassing sites and implement actions to mitigate the risks.

Alongside further research, authorities need to implement regulations and legislation for “gas-resistant” codes, land-use planning and definition of air quality standards. Investment in public policies to reduce risks is thus crucial in any volcanic area prone to be affected by diffuse degassing phenomena.

## Acknowledgments

This study was partially funded by the Portuguese Science Foundation (FTC) through the project SONDA - Synchronous Oceanic and Atmospheric Data Acquisition (PTDC/EME-SIS/1960/2020). The authors sincerely thank the editor and three anonymous reviewers who provided constructive feedback and contributed to improving significantly the manuscript. Vittorio Zanon helped with the graphic figure and Hadi Cruz with the English.

## Author contributions

F.V. and C.S. conceptualized the study. F.V. collected the data, applied the methodology including the formal data analysis and wrote the original draft. C.S. revised and edited the final manuscript for submission.

## Declaration of interests

The authors declare no competing interests.

## Supporting citations

The following references appear in the Supplemental Information: ^87^

## Supplemental information

Document S1. Figure S1

Table S1. Search strategy applied on the five databases

Table S2. Data extracted from the selected peer-reviewed articles with the main impacts and mitigation strategies applied on diffuse CO_2_ degassing zones (n.m. – not measured; n.e. – not explored) All concentration values were converted in ppm, and soil CO_2_ fluxes into g m^−2^ d^−1^. Soil gas concentrations were measured at different depths (from 0.2 m to about 1 m depth).
