# Supplementary material for: Hazardous volcanic CO2 diffuse degassing areas – A systematic review on environmental impacts, health, and mitigation strategies
Source: iScience. 2024 Sep 19;27(10):110990. doi: 10.1016/j.isci.2024.110990 (PMC11490718; doi:10.1016/j.isci.2024.110990)
Supplement: Document S1. Figure S1 [file mmc1.pdf]

iScience, Volume *27*

Supplemental information

Hazardous volcanic CO_2 diffuse degassing areas – A systematic review on environmental impacts, health, and mitigation strategies

Fátima Viveiros and Catarina Silva

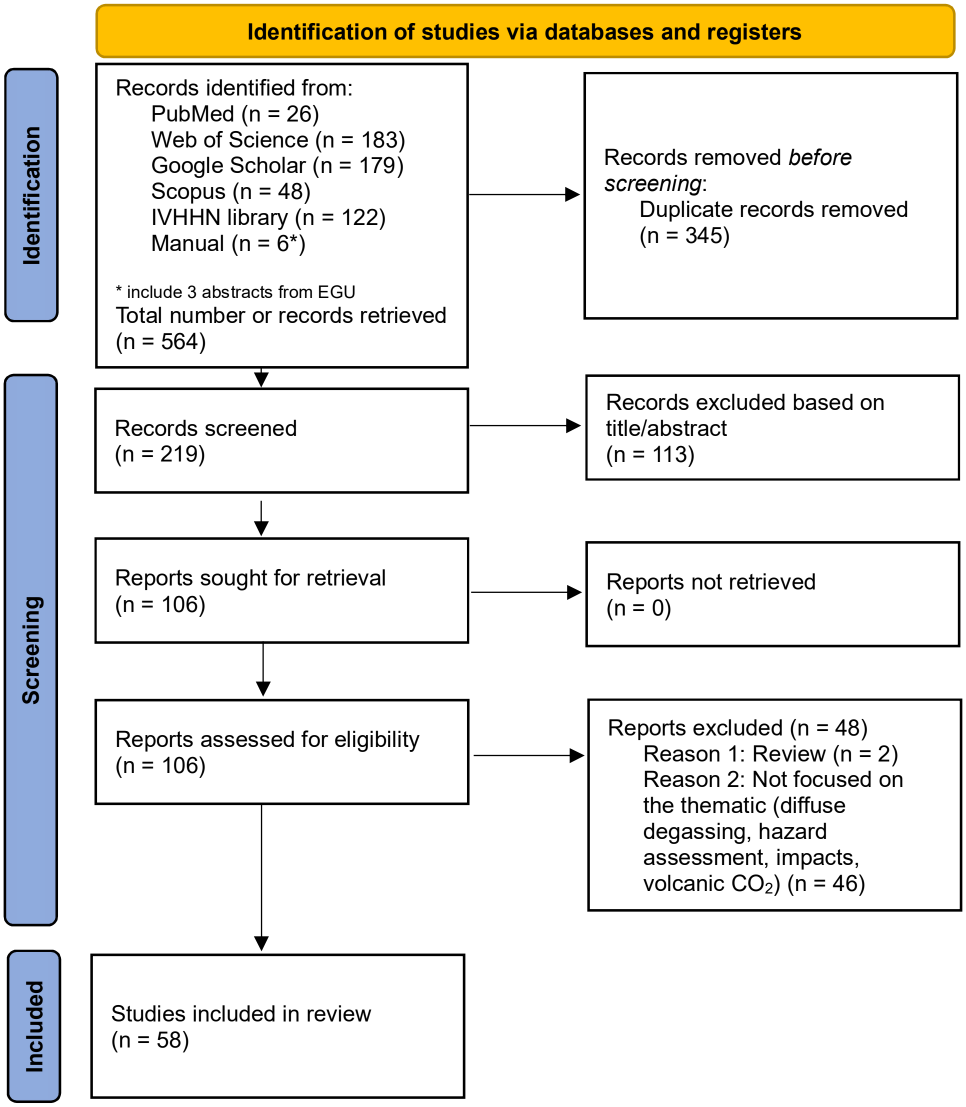

Figure S1 – Number of scientific articles included in the review, based on the PRISMA[87] strategy.
