# Supplementary material for: Hazardous volcanic CO2 diffuse degassing areas – A systematic review on environmental impacts, health, and mitigation strategies
Source: iScience. 2024 Sep 19;27(10):110990. doi: 10.1016/j.isci.2024.110990 (PMC11490718; doi:10.1016/j.isci.2024.110990)
Supplement: Table S1. Search strategy applied on the five databases [file mmc2.pdf]

Table S1 – Search strategy applied on the five databases.

Databases	Search Strategy	Fields
PubMed, Web of Science	(diffuse soil CO_2 degassing) AND (environment)	All Fields; Any Field
	(diffuse soil CO_2 degassing) AND (impacts)	
	(diffuse soil CO_2 degassing) AND (health)	
	(diffuse soil CO_2 degassing) AND (hazard)	
Google Scholar, Scopus	"diffuse degassing" AND CO_2 AND soil AND ("health" AND "environment" AND "impact" AND "hazard")	All Fields
IVHHN Library	Air quality<br>Animal health<br>Evacuation and displacement<br>Gas and aerosols hazards and impacts – respiratory<br>Geothermal gases & water<br>Risk assessment	Category
