# Supplementary material for: Hazardous volcanic CO2 diffuse degassing areas – A systematic review on environmental impacts, health, and mitigation strategies
Source: iScience. 2024 Sep 19;27(10):110990. doi: 10.1016/j.isci.2024.110990 (PMC11490718; doi:10.1016/j.isci.2024.110990)
Supplement: Data S1. Methods [file mmc4.pdf]

STAR Methods

RESOURCE AVAILABILITY

Lead contact

Further information and requests about the current review should be directed to and will be fulfilled by the lead contact, Fátima Viveiros (maria.fb.viveiros@azores.gov.pt).

Materials availability

This study did not generate new reagents.

Data and code availability

This paper analyses existing, publicly available data. These accession numbers for the datasets are listed in the key resources table. This paper does not report original code. Any additional information required to reanalyse the data reported in this paper is available from the lead contact upon request.

METHOD DETAILS

A systematic review was conducted in order to identify studies carried out in hazardous volcanic CO_2 diffuse degassing areas and that focus essentially on impacts on environment (animals, vegetation, soils, microbiota), infrastructure and on population (health). The review followed the PRISMA guidelines[87] and five databases (PubMed, Web of Science, Google Scholar, Scopus, and the International Volcanic Health Hazard Network - IVHHN library) were searched (Figure S1, Supplemental Material). Inclusion criteria: only peer-reviewed articles written in English, and no constrains on time were defined. Excluded criteria: non-volcanic regions and lakes CO_2 degassing sites were not considered.

The search strategy included multiple combinations of the terms/keywords “diffuse soil CO_2 degassing”, “impacts”, “health”, “environment” and “hazard” (Table S1, Supplemental Material). Retrieved articles were imported into a reference management *software* (*EndNote 20*), and duplicates were removed. Articles were first screened based on the abstract and titles, and then full-text reading was done on the articles identified as eligible. During the screening, and based on the references found out in the included articles, three articles were manually included in the review. Considering that some recent study cases (e.g., 2021 Tajogaite eruption) are not still

available as peer-reviewed article, abstracts written in English and available in the web platforms were also screened in order to complement and update this study. Three abstracts presented in the EGU 2023 meeting[61,62,63] were then used to report the information related with the 2021 Tajogaite eruption in La Palma.

Data were extracted to a table (Table S2, Supplemental Material) in order to evaluate the main outcomes of the different studies, especially characterize the study sites (volcanic systems, type of activity), report main hazards (air/soil CO_2 concentrations/fluxes), identify vulnerable structures and the type of impact (health, environment), as well as the potential mitigation strategies to reduce the risk.
